# Supplementary material for: Quality of life and multiple long-term conditions in Southeast Asia: a systematic review and meta-analysis
Source: Nat Commun. 2026 Jan 26;17:1461. doi: 10.1038/s41467-025-68197-z (PMC12886794; doi:10.1038/s41467-025-68197-z)
Supplement: Supplementary file 1 — Supplementary Information [file 41467_2025_68197_MOESM1_ESM.pdf]

Quality of life and multiple long-term conditions in Southeast Asia: a systematic review and meta-analysis

Supplementary Table 1. Characteristics of included studies

First Author (Year)	Country	Study Design (Sample Size)	Total sample size (MLTC only)	Mean age	Sex in %	Eligible conditions (%)	QOL Tool (Version)	QOL Outcome (Study rating)	ROB Score
Alfian et al., 2021[1]	Indonesia	Multi-center cross sectional	503 (439)	NR	M- 32.4<br>F- 67.6	T2D (100)<br>Hypertension (87.3)	EQ-5D-5L<br>(Indonesia)	0.80±0.20<br>(Good)	Good
Banerjee et al., 2022[2]	India	Mixed methods	50 (NR)	56.1 ± 8.96	M- 74<br>F- 26	Parkinson's disease (100)<br>Depression (>80)<br>Anxiety (>80)	PDQ-8	44.90±15.33<br>(Poor)	Poor
Barne et al., 2024[3]	India	Cross-sectional	32 (24)	35.4±5.73	M- 50<br>F- 50	Pulmonary tuberculosis (100)<br>Anaemia (75)	SQRQ	69.57±17.72<br>(Poor)	Fair
Basu et al., 2021[4]	India	Cross-sectional survey	219 (165)	NR	NR	Diabetes (100)<br>Depression (75.2)	WHOQOL-BREF (Hindi)	75.31 (Good)	Fair
Dejvorakul et al., 2019[5]	Thailand	Cross-sectional interviews	188 (160)	NR	M: 57<br>F: 43	End-stage renal disease (100)<br>Hypertension (85)	KDQOL-SF (Thai)	NR	Fair
Fadhil et al., 2020[6]	Thailand	Randomised controlled trial	53 (53)	64.8±13.4	M: 41<br>F: 59	Major depressive disorder (100)<br>CKD (100)	WHOQOL-BREF	Agomelatine<br>72.40±12.40<br>Setraline<br>70.7±9.9	Good
Gautam et al., 2023	Nepal	Cross-sectional observational	105 (105)	49.4± 11.11	M: 49.5<br>F: 50.5	Diabetes (100)<br>Hypertension (100)	EQ-5D-5L<br>EQ VAS	EQ-5D-5L<br>0.78± 0.09<br>(Good)<br>EQ VAS<br>58.43 ± 10.88	Fair
Gupta et al., 2024[7]	India	Cross-sectional	110 (110)	52.6±12.2	M: 57.3<br>F: 42.7	Glaucoma (100)<br>Depression (100)<br>Anxiety (100)	GQL-15	NR	Good
Hanspal et al., 2021[8]	India	Cross-sectional	160 (160)	41.77 ± 8.11	M: 78<br>F: 22	End stage renal disease (100)<br>Diabetes (100)	EQ-5D-3L	NR	Good
Hussain et al., 2019[9]	India	Cross-sectional	323 (323)	56 ± 11.25	M: 49.4<br>F: 51.7	T2D (100)<br>CKD (100)<br>Anaemia (70.3)	EQ-5D-3L	NR	Good
Koesoemadinata et al., 2021[10]	Indonesia	Randomised controlled trial	108 (108)	NR	Intervention<br>M: 48.3<br>F: 51.7<br>Control<br>M: 45.8<br>F: 54.2	T2D (100)<br>TB (100)	EQ-VAS	Intervention<br>70.00±16.10<br>Control<br>67.5±15.1	Poor

First Author (Year)	Country	Study Design (Sample Size)	Total sample size (MLTC only)	Mean age	Sex in %	Eligible conditions (%)	QOL Tool (Version)	QOL Outcome (Study rating)	ROB Score
Komariah et al., 2023[11]	Indonesia	Quasi-experimental	40 (28)	NR	M: 50<br>F: 50	CKD (100)<br>Hypertension (70)	KDQOL (Indonesia)	Intervention<br>47.25±7.29<br>Control<br>48.29±7.09	Fair
Kumar et al., 2021[12]	India	Cross-sectional	120 (95)	50.6 ± 12.6	M: 64.2<br>F: 35.8	End stage renal disease (100)<br>Hypertension (79.1)	KDQOL-SF (Tamil)	NR	Fair
Kunwar et al., 2020[13]	Nepal	Cross-sectional	143 (108)	45.8	M: 64<br>F: 36	CKD (100)<br>Depression (75.5)	WHOQOL-8 (Nepali)	NR	Good
Kuptniratsaikul et al., 2009[14]	Thailand	Multi-center, prospective cohort	327 (245)	62± 12	M: 59<br>W: 41	Stroke (100)<br>Hypertension (74.9)	WHOQOL-BREF (Thai)	NR	Fair
Manimmanakorn et al., 2008[15]	Thailand	Multi-center, prospective analytical	203 (163)	60.9±12.0	M: 60.6<br>F: 39.4	Stroke (100)<br>Hypertension (80.3)	WHOQOL-BREF	NR	Fair
Mishra et al., 2020[16]	India	Prospective non-interventional	96 (96)	Metformin users<br>47.6 ± 6.3<br>Metformin non users<br>49.0± 5.8	Metformin users<br>M: 62.5<br>F: 37.5<br>Metformin non users<br>M: 54.1<br>F: 45.9	T2D (100)<br>Pulmonary TB (100)	DR-12 (Indian)	NR	Fair
Modi et al., 2020[17]	India	Prospective cohort	2919 (2101)	50±12	M: 69<br>F: 31	CKD (100)<br>Hypertension (72)	KDQOL-36	NR	Fair
Murali et al., 2015[18]	India	Observational and prospective	50 (39)	55.8±10.3	M: 58<br>F: 42	End-stage renal disease (100)<br>Diabetes Mellitus (78)	KDQOL-SF	NR	Fair
Patel et al., 2014[19]	India	Cross-sectional	114 (80)	56.8±10.5	M:42.1<br>F: 57.9	T2D (100)<br>Hypertension (70.2)	WHOQOL-BREF<br>ADS (Gujarati)	WHOQOL-BREF<br>76.65±8.29 (Good)<br>ADS<br>19.90±3.40 (Average)	Good
Prakash et al., 2019[20]	India	Cross-sectional	210 (186)	NR	F: 100	Breast cancer (100)<br>Anxiety (88.5)	EORTC QLQ	NR	Fair
Prasad et al., 2022[21]	India	Prospective cross-sectional study	2880 (2880)	NR	M: 56.25<br>F: 43.75	Diabetes (100)<br>Hypertension (100)	SF-36	NR	Fair
Rahman et al., 2024[22]	Bangladesh	Cross-sectional	197 (197)	48.1±13.8	M: 76<br>F: 24	Metastatic cancer (100)<br>Depression (73.1)	FACT-G scale (Bengali)	46.00±10.70 (Poor)	Fair

First Author (Year)	Country	Study Design (Sample Size)	Total sample size (MLTC only)	Mean age	Sex in %	Eligible conditions (%)	QOL Tool (Version)	QOL Outcome (Study rating)	ROB Score
Ranabhat et al., 2020[23]	Nepal	Cross-sectional comparative study design	161 (124)	40.7±12.0	M: 75.2<br>F: 24.8	Chronic kidney disease (100)<br>Hypertension (77)	WHOQOL-BREF (Nepali)	12.43±1.63 (Average)	Fair
Saisunantararom et al., 2015[24]	Thailand	cross-sectional descriptive study	63 (49)	64±9.1	M: 20.6<br>F:79.4	CKD (100)<br>Diabetes Mellitus (77.8)	9-THAI (Thai)	NR	Fair
Singh et al., 2019[25]	India	Pretest-posttest	82 (82)	NR	NR	Diabetes Mellitus (100)<br>Chronic Heart Disease (100)	SF-36<br>SAQ	NR	Good
Singh et al., 2021[26]	India	Cross-sectional	88 (68)	47.7±14.6	M: 14<br>F: 86	Rheumatoid arthritis (100)<br>Depression (77)	WHOQOL-BREF (Hindi)	NR	Fair
Sridhar et al., 2024[27]	India	Prospective observational	196 (196)	NR	NR	Hypertension (100)<br>Stroke (100)	SS-QOL	NR	Fair
Sudarisan et al., 2019[28]	India	Cross-sectional	234 (164)	57.4±10.8	M: 40.2<br>F: 59.8	Cancer (100)<br>Depression (70)	WHOQOL-BREF (Tamil)	NR	Fair
Sukcharoen et al., 2024 [29]	Thailand	Cross-sectional	252 (207)	68.9±6.6	M: 57.1<br>F: 42.9	T2D (100)<br>Hypertension (82.1)	D-39	NR	Fair
Thanakiatpinyo et al., 2014[30]	Thailand	Randomised controlled trial	50 (50)	Traditional Thai massage (TTM) group 60.0±6.9<br>Physical therapy (PT) group<br>65.8±8.1	Traditional Thai massage group<br>M: 91.7<br>F: 8.3<br>Physical therapy group<br>M: 57.7<br>F: 42.3	Stroke (100)<br>TTM: Hypertension (95.8)<br>PT: Hypertension (88.5)	Pictorial Thai QoL Test (Thai)	Traditional Thai massage group<br>41.10±15.60<br>Physical therapy group<br>42.8±13.70	Good
Thancharoen et al., 2020[31]	Thailand	Cross-sectional survey	379 (331)	65.7±12.2	M: 52.8<br>F: 47.2	CKD (100)<br>Hypertension (87.3)	EQ-5D-5L (Thai)	Cognitive impairment<br>0.78± 0.16<br>Normal cognition<br>0.85± 0.18 (Good)	Fair
Tungsirikoon et al., 2023[32]	Thailand	Cross-sectional	335 (245)	62.5 ± 11.8	M:32.2<br>F: 67.8	T2D (100)<br>Hypertension (73.8)	ADDQoL (Thai)	NR	Fair
Yapa et al., 2023[33]	Sri Lanka	Cross-sectional	886 (626)	57.1±11.1	M: 68.4<br>F: 31.6	CKD (100)<br>Hypertension (70.6)	EQ-5D-3L (Sinhalese)	0.57±0.32 (Average)	Good

NR = Not reported; T2D = Type 2 Diabetes; CKD = Chronic kidney disease; TB = Tuberculosis

Supplementary Table 2. MLTC combination

Physical + Physical	Physical + Mental	NCDs + Infectious	Infectious + Infectious
Diabetes + hypertension [1, 19, 21, 29, 32, 34]	Parkinson's disease + depression + anxiety [2]	Diabetes + TB [10, 16]	Pulmonary tuberculosis + anaemia [3]
ESRD + hypertension [5, 12]	Diabetes + depression [4]		
ESRD + diabetes [8, 18]	CKD + major depressive disorder [6]		
CKD + T2D + Anaemia [9]	CKD + depression [13]		
Hypertension + Stroke [14, 15, 27, 30]	Breast cancer + anxiety [20]		
CKD + hypertension [11, 17, 23, 31, 33]	Rheumatoid arthritis + depression [26]		
CKD + diabetes [24]	Cancer + depression [22, 28]		
Chronic heart disease + diabetes [25]	Glaucoma + depression + anxiety [7]		

MLTC = multiple long-term conditions; NCD = non-communicable disease; ESRD = end-stage kidney disease; CKD = chronic kidney disease; TB = tuberculosis

Supplementary Table 3: PRISMA Checklist

Section and Topic	Item #	Checklist item	Location where item is reported
**TITLE**			
Title	1	Identify the report as a systematic review.	Page 1
**ABSTRACT**			
Abstract	2	See the PRISMA 2020 for Abstracts checklist.	Page 1
**INTRODUCTION**			
Rationale	3	Describe the rationale for the review in the context of existing knowledge.	Pages 2, 3
Objectives	4	Provide an explicit statement of the objective(s) or question(s) the review addresses.	Pages 2, 3
**METHODS**			
Eligibility criteria	5	Specify the inclusion and exclusion criteria for the review and how studies were grouped for the syntheses.	Pages 10 -11
Information sources	6	Specify all databases, registers, websites, organisations, reference lists and other sources searched or consulted to identify studies. Specify the date when each source was last searched or consulted.	Pages 10 -11
Search strategy	7	Present the full search strategies for all databases, registers and websites, including any filters and limits used.	Supplementary Table 5
Selection process	8	Specify the methods used to decide whether a study met the inclusion criteria of the review, including how many reviewers screened each record and each report retrieved, whether they worked independently, and if applicable, details of automation tools used in the process.	Page 10
Data collection process	9	Specify the methods used to collect data from reports, including how many reviewers collected data from each report, whether they worked independently, any processes for obtaining or confirming data from study investigators, and if applicable, details of automation tools used in the process.	Page 11
Data items	10a	List and define all outcomes for which data were sought. Specify whether all results that were compatible with each outcome domain in each study were sought (e.g. for all measures, time points, analyses), and if not, the methods used to decide which results to collect.	Page 11
	10b	List and define all other variables for which data were sought (e.g. participant and intervention characteristics, funding sources). Describe any assumptions made about any missing or unclear information.	Page 11
Study risk of bias assessment	11	Specify the methods used to assess risk of bias in the included studies, including details of the tool(s) used, how many reviewers assessed each study and whether they worked independently, and if applicable, details of automation tools used in the process.	Page 11
Effect measures	12	Specify for each outcome the effect measure(s) (e.g. risk ratio, mean difference) used in the synthesis or presentation of results.	N/A
Synthesis methods	13a	Describe the processes used to decide which studies were eligible for each synthesis (e.g. tabulating the study intervention characteristics and comparing against the planned groups for each synthesis (item #5)).	Page 11
	13b	Describe any methods required to prepare the data for presentation or synthesis, such as handling of missing summary statistics, or data conversions.	Page 11
	13c	Describe any methods used to tabulate or visually display results of individual studies and syntheses.	Page 11
	13d	Describe any methods used to synthesize results and provide a rationale for the choice(s). If meta-analysis was performed, describe the model(s), method(s) to identify the presence and extent of statistical heterogeneity, and software package(s) used.	Page 11

Section and Topic	Item #	Checklist item	Location where item is reported
	13e	Describe any methods used to explore possible causes of heterogeneity among study results (e.g. subgroup analysis, meta-regression).	Page 11
	13f	Describe any sensitivity analyses conducted to assess robustness of the synthesized results.	Page 11
Reporting bias assessment	14	Describe any methods used to assess risk of bias due to missing results in a synthesis (arising from reporting biases).	N/A
Certainty assessment	15	Describe any methods used to assess certainty (or confidence) in the body of evidence for an outcome.	N/A
**RESULTS**			
Study selection	16a	Describe the results of the search and selection process, from the number of records identified in the search to the number of studies included in the review, ideally using a flow diagram.	Page 3, Figure 1
	16b	Cite studies that might appear to meet the inclusion criteria, but which were excluded, and explain why they were excluded.	Figure 1
Study characteristics	17	Cite each included study and present its characteristics.	Page 3, Supplementary Table 1
Risk of bias in studies	18	Present assessments of risk of bias for each included study.	Page 3, Supplementary Table 1
Results of individual studies	19	For all outcomes, present, for each study: (a) summary statistics for each group (where appropriate) and (b) an effect estimate and its precision (e.g. confidence/credible interval), ideally using structured tables or plots.	Page 5, Figure 2
Results of syntheses	20a	For each synthesis, briefly summarise the characteristics and risk of bias among contributing studies.	Supplementary Table 1
	20b	Present results of all statistical syntheses conducted. If meta-analysis was done, present for each the summary estimate and its precision (e.g. confidence/credible interval) and measures of statistical heterogeneity. If comparing groups, describe the direction of the effect.	Pages 3-7
	20c	Present results of all investigations of possible causes of heterogeneity among study results.	Pages 3-7
	20d	Present results of all sensitivity analyses conducted to assess the robustness of the synthesized results.	Page 4
Reporting biases	21	Present assessments of risk of bias due to missing results (arising from reporting biases) for each synthesis assessed.	N/A
Certainty of evidence	22	Present assessments of certainty (or confidence) in the body of evidence for each outcome assessed.	N/A
**DISCUSSION**			
Discussion	23a	Provide a general interpretation of the results in the context of other evidence.	Pages 7-10
	23b	Discuss any limitations of the evidence included in the review.	Page 9
	23c	Discuss any limitations of the review processes used.	Page 9
	23d	Discuss implications of the results for practice, policy, and future research.	Pages 9-10
**OTHER INFORMATION**			

Section and Topic	Item #	Checklist item	Location where item is reported
Registration and protocol	24a	Provide registration information for the review, including register name and registration number, or state that the review was not registered.	Page 11
	24b	Indicate where the review protocol can be accessed, or state that a protocol was not prepared.	Page 11
	24c	Describe and explain any amendments to information provided at registration or in the protocol.	N/A
Support	25	Describe sources of financial or non-financial support for the review, and the role of the funders or sponsors in the review.	Page 16
Competing interests	26	Declare any competing interests of review authors.	Online submission system and page 16
Availability of data, code and other materials	27	Report which of the following are publicly available and where they can be found: template data collection forms; data extracted from included studies; data used for all analyses; analytic code; any other materials used in the review.	Page 12

Supplementary Table 4: SWiM Checklist

**SWiM is intended to complement and be used as an extension to PRISMA**			
**SWiM reporting item**	**Item description**	**Page in manuscript where item is reported**	**Other***
Methods			
**1** Grouping studies for synthesis	1a) Provide a description of, and rationale for, the groups used in the synthesis (e.g., groupings of populations, interventions, outcomes, study design)	Pages 10-11	Table 1
	1b) Detail and provide rationale for any changes made subsequent to the protocol in the groups used in the synthesis	N/A	
**2** Describe the standardised metric and transformation methods used	Describe the standardised metric for each outcome. Explain why the metric(s) was chosen, and describe any methods used to transform the intervention effects, as reported in the study, to the standardised metric, citing any methodological guidance consulted	N/A	
**3** Describe the synthesis methods	Describe and justify the methods used to synthesise the effects for each outcome when it was not possible to undertake a meta-analysis of effect estimates	Page 11	
**4** Criteria used to prioritise results for summary and synthesis	Where applicable, provide the criteria used, with supporting justification, to select the particular studies, or a particular study, for the main synthesis or to draw conclusions from the synthesis (e.g., based on study design, risk of bias assessments, directness in relation to the review question)	Page 11	Table 1
**SWiM reporting item**	**Item description**	**Page in manuscript where item is reported**	**Other***
**5** Investigation of heterogeneity in reported effects	State the method(s) used to examine heterogeneity in reported effects when it was not possible to undertake a meta-analysis of effect estimates and its extensions to investigate heterogeneity	Page 10	
**6** Certainty of evidence	Describe the methods used to assess certainty of the synthesis findings	N/A	
**7** Data presentation methods	Describe the graphical and tabular methods used to present the effects (e.g., tables, forest plots, harvest plots). Specify key study characteristics (e.g., study design, risk of bias) used to order the studies, in the text and any tables or graphs, clearly referencing the studies included	Figure 1	
Results			
**8** Reporting results	For each comparison and outcome, provide a description of the synthesised findings, and the certainty of the findings. Describe the result in language that is consistent with the question the synthesis addresses, and indicate which studies contribute to the synthesis	Pages 10-11	
Discussion			
**9** Limitations of the synthesis	Report the limitations of the synthesis methods used and/or the groupings used in the synthesis, and how these affect the conclusions that can be drawn in relation to the original review question	Pages 7-10	

*If the information is not provided in the systematic review, give details of where this information is available (e.g., protocol, other published papers (provide citation details), or website (provide the URL)).

Supplementary Figure 1. Mapping of QOL domains

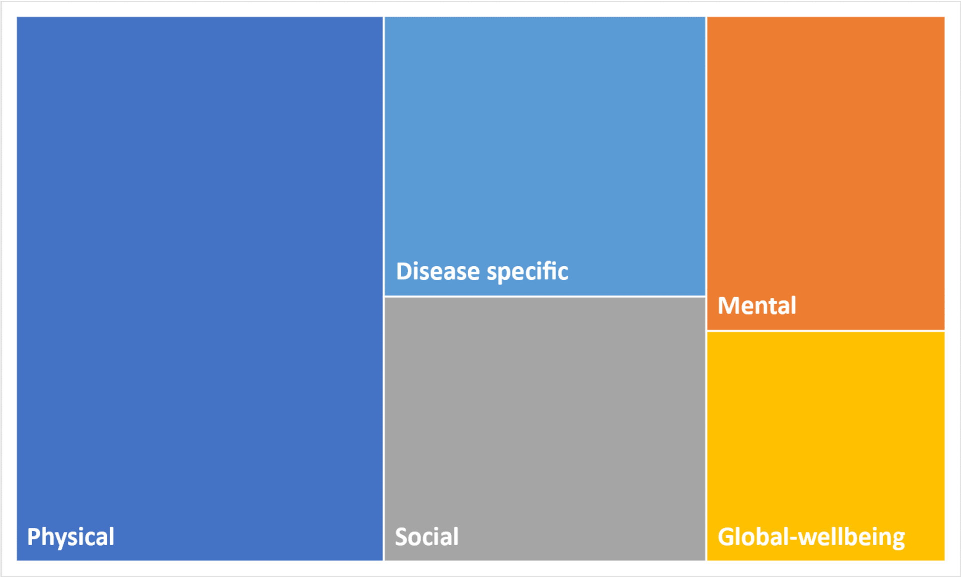

Supplementary Table 5. Full Search Strategy for OVID MEDLINE

Ovid MEDLINE Search Strategy

1 multimorbidity.mp. or exp Multimorbidity/ 7675
2 ("multiple long-term conditions" or "multiple long term conditions" or MLTC).mp. [mp=title, book title, abstract, original title, name of substance word, subject heading word, floating sub-heading word, keyword heading word, organism supplementary concept word, protocol supplementary concept word, rare disease supplementary concept word, unique identifier, synonyms, population supplementary concept word, anatomy supplementary concept word] 479
3 "concurrent conditions".mp. [mp=title, book title, abstract, original title, name of substance word, subject heading word, floating sub-heading word, keyword heading word, organism supplementary concept word, protocol supplementary concept word, rare disease supplementary concept word, unique identifier, synonyms, population supplementary concept word, anatomy supplementary concept word] 260
4 comorbidity.mp. or Comorbidity/ 177929
5 (co-morbid* or comorbid*).mp. [mp=title, book title, abstract, original title, name of substance word, subject heading word, floating sub-heading word, keyword heading word, organism supplementary concept word, protocol supplementary concept word, rare disease supplementary concept word, unique identifier, synonyms, population supplementary concept word, anatomy supplementary concept word] 321190
6 cardiovascular disease.mp. or Cardiovascular Diseases/ 276671
7 stroke.mp. or Stroke/ 363156
8 coronary artery disease.mp. or Coronary Artery Disease/ 140897
9 heart failure.mp. or Heart Failure/ 249007
10 peripheral artery disease.mp. or Peripheral Arterial Disease/ 15119
11 "peripheral arterial disease".mp. [mp=title, book title, abstract, original title, name of substance word, subject heading word, floating sub-heading word, keyword heading word, organism supplementary concept word, protocol supplementary concept word, rare disease supplementary concept word, unique identifier, synonyms, population supplementary concept word, anatomy supplementary concept word] 18539
12 heart valve.mp. or Heart Valves/ 82424
13 arrhythmia.mp. or Arrhythmias, Cardiac/ 125987
14 atrial fibrillation.mp. or Atrial Fibrillation/ 102837
15 Venous Thromboembolism/ or venous thromboembolic disease.mp. or Venous Thrombosis/ 43307
16 exp Aneurysm/ or aneurysm.mp. 167397
17 hypertension.mp. or exp Hypertension/ 555838
18 "high blood pressure".mp. [mp=title, book title, abstract, original title, name of substance word, subject heading word, floating sub-heading word, keyword heading word, organism supplementary concept word, protocol supplementary concept word, rare disease supplementary concept word, unique identifier, synonyms, population supplementary concept word, anatomy supplementary concept word] 17556
19 exp Diabetes Mellitus, Type 2/ or exp Diabetes Complications/ or exp Diabetes Mellitus, Type 1/ or diabetes.mp. 776490

20 addison disease.mp. or Addison Disease/ 4938
21 cystic fibrosis.mp. or Cystic Fibrosis/ 57110
22 thyroid disease.mp. or exp Thyroid Diseases/ 165121
23 chronic obstructive pulmonary disease.mp. or Pulmonary Disease, Chronic Obstructive/ 76658
24 COPD.mp. [mp=title, book title, abstract, original title, name of substance word, subject heading word, floating sub-heading word, keyword heading word, organism supplementary concept word, protocol supplementary concept word, rare disease supplementary concept word, unique identifier, synonyms, population supplementary concept word, anatomy supplementary concept word] 57396
25 Asthma/ or asthma.mp. 195863
26 bronchiectasis.mp. or Bronchiectasis/ 14417
27 parkinson disease.mp. or Parkinson Disease/ 89562
28 Epilepsy/ or epilepsy.mp. 160668
29 multiple sclerosis.mp. or Multiple Sclerosis/ 97732
30 Paralysis/ or paralysis.mp. 75885
31 transient ischemic attack.mp. or Ischemic Attack, Transient/ 28139
32 ("transient ischaemic attack" or TIA).mp. [mp=title, book title, abstract, original title, name of substance word, subject heading word, floating sub-heading word, keyword heading word, organism supplementary concept word, protocol supplementary concept word, rare disease supplementary concept word, unique identifier, synonyms, population supplementary concept word, anatomy supplementary concept word] 11526
33 peripheral neuropathy.mp. or Peripheral Nervous System Diseases/ 40099
34 chronic pain.mp. or Chronic Pain/ 57094
35 cancer.mp. or Neoplasms/ 2335melan999
36 (melanoma or benign or malignant or tumour).mp. [mp=title, book title, abstract, original title, name of substance word, subject heading word, floating sub-heading word, keyword heading word, organism supplementary concept word, protocol supplementary concept word, rare disease supplementary concept word, unique identifier, synonyms, population supplementary concept word, anatomy supplementary concept word] 925792
37 dementia.mp. or Dementia/ 155906
38 Schizophrenia/ or schizophrenia.mp. 158558
39 exp Depression/ or depression.mp. 483278
40 exp Anxiety/ or anxiety.mp. 303243
41 bipolar.mp. or Bipolar Disorder/ 89678
42 drug abuse.mp. or Substance-Related Disorders/ 115380
43 alcohol abuse.mp. or Alcoholism/ 89837
44 eating disorder.mp. or "Feeding and Eating Disorders"/ 26317
45 autism.mp. or Autistic Disorder/ 65656
46 autistic.mp. [mp=title, book title, abstract, original title, name of substance word, subject heading word, floating sub-heading word, keyword heading word, organism supplementary concept word, protocol supplementary concept word, rare disease supplementary concept word, unique identifier, synonyms, population supplementary concept word, anatomy supplementary concept word] 31712
47 post-traumatic stress disorder.mp. or Stress Disorders, Post-Traumatic/ 47223
48 PTSD.mp. [mp=title, book title, abstract, original title, name of substance word, subject heading word, floating sub-heading word, keyword heading word, organism supplementary concept word, protocol supplementary concept word, rare disease supplementary concept word, unique identifier, synonyms, population supplementary concept word, anatomy supplementary concept word] 32686
49 connective tissue disease.mp. or Connective Tissue Diseases/ 13739

50 exp Arthritis/ or arthritis.mp. 357232
51 osteoarthritis.mp. or Osteoarthritis/ 109013
52 Musculoskeletal Diseases/ or musculoskeletal.mp. 83824
53 Osteoporosis/ or osteoporosis.mp. 100104
54 Gout/ or gout.mp. 19621
55 liver disease.mp. or Liver Diseases/ 167649
56 inflammatory bowel disease.mp. or Inflammatory Bowel Diseases/62721
57 pancreatic disease.mp. or Pancreatic Diseases/ 16922
58 pancreatitis.mp. or Pancreatitis/ 76674
59 peptic ulcer.mp. or Peptic Ulcer/ 51597
60 chronic kidney disease.mp. or exp Renal Insufficiency, Chronic/ 168324
61 Kidney Failure, Chronic/ or renal disease.mp. 143627
62 (end stage or dialysis or transplant or renal failure).mp. [mp=title, book title, abstract, original title, name of substance word, subject heading word, floating sub-heading word, keyword heading word, organism supplementary concept word, protocol supplementary concept word, rare disease supplementary concept word, unique identifier, synonyms, population supplementary concept word, anatomy supplementary concept word] 483791
63 endometriosis.mp. or Endometriosis/ 32563
64 urinary tract infection.mp. or Urinary Tract Infections/ 56831
65 anemia.mp. or exp Anemia/ 234897
66 anaemia.mp. 36988
67 Cataract/ or cataract.mp. 72592
68 Visually Impaired Persons/ or Vision Disorders/ or vision impairment.mp. or Blindness/ 53197
69 (blind or blindness).mp. [mp=title, book title, abstract, original title, name of substance word, subject heading word, floating sub-heading word, keyword heading word, organism supplementary concept word, protocol supplementary concept word, rare disease supplementary concept word, unique identifier, synonyms, population supplementary concept word, anatomy supplementary concept word] 337269
70 hearing loss.mp. or Hearing Loss/ 78073
71 ("hearing impairment" or "hearing impaired" or deaf).mp. [mp=title, book title, abstract, original title, name of substance word, subject heading word, floating sub-heading word, keyword heading word, organism supplementary concept word, protocol supplementary concept word, rare disease supplementary concept word, unique identifier, synonyms, population supplementary concept word, anatomy supplementary concept word] 28830
72 meniere disease.mp. or Meniere Disease/ 7762
73 exp HIV/ or HIV.mp. 397411
74 "human immunodeficiency virus".mp. [mp=title, book title, abstract, original title, name of substance word, subject heading word, floating sub-heading word, keyword heading word, organism supplementary concept word, protocol supplementary concept word, rare disease supplementary concept word, unique identifier, synonyms, population supplementary concept word, anatomy supplementary concept word] 107261
75 AIDS.mp. or Acquired Immunodeficiency Syndrome/ 235204
76 "acquired immunodeficiency syndrome".mp. [mp=title, book title, abstract, original title, name of substance word, subject heading word, floating sub-heading word, keyword heading word, organism supplementary concept word, protocol supplementary concept word, rare disease supplementary concept word, unique identifier, synonyms, population supplementary concept word, anatomy supplementary concept word] 92556

77 tuberculosis.mp. or exp Tuberculosis/ 277103
78 lyme disease.mp. or Lyme Disease/ 13968
79 covid-19.mp. or exp COVID-19/ 331325
80 congenital.mp. [mp=title, book title, abstract, original title, name of substance word, subject heading word, floating sub-heading word, keyword heading word, organism supplementary concept word, protocol supplementary concept word, rare disease supplementary concept word, unique identifier, synonyms, population supplementary concept word, anatomy supplementary concept word] 389242
81 1 or 2 or 3 or 4 or 5 or 6 or 7 or 8 or 9 or 10 or 11 or 12 or 13 or 14 or 15 or 16 or 17 or 18 or 19 or 20 or 21 or 22 or 23 or 24 or 25 or 26 or 27 or 28 or 29 or 30 or 31 or 32 or 33 or 34 or 35 or 36 or 37 or 38 or 39 or 40 or 41 or 42 or 43 or 44 or 45 or 46 or 47 or 48 or 49 or 50 or 51 or 52 or 53 or 54 or 55 or 56 or 57 or 58 or 59 or 60 or 61 or 62 or 63 or 64 or 65 or 66 or 67 or 68 or 69 or 70 or 71 or 72 or 73 or 74 or 75 or 76 or 77 or 78 or 79 or 80 10156026
82 quality of life.mp. or "Quality of Life"/ 438868
83 (QOL or "life quality").mp. [mp=title, book title, abstract, original title, name of substance word, subject heading word, floating sub-heading word, keyword heading word, organism supplementary concept word, protocol supplementary concept word, rare disease supplementary concept word, unique identifier, synonyms, population supplementary concept word, anatomy supplementary concept word] 60282
84 ("quality of life scale" or QOLS).mp. [mp=title, book title, abstract, original title, name of substance word, subject heading word, floating sub-heading word, keyword heading word, organism supplementary concept word, protocol supplementary concept word, rare disease supplementary concept word, unique identifier, synonyms, population supplementary concept word, anatomy supplementary concept word] 3399
85 ("World Health Organization Quality of Life Instrument" or WHOQOL).mp. [mp=title, book title, abstract, original title, name of substance word, subject heading word, floating sub-heading word, keyword heading word, organism supplementary concept word, protocol supplementary concept word, rare disease supplementary concept word, unique identifier, synonyms, population supplementary concept word, anatomy supplementary concept word] 3957
86 ("McGill Quality of Life Questionnaire" or MQLQ).mp. [mp=title, book title, abstract, original title, name of substance word, subject heading word, floating sub-heading word, keyword heading word, organism supplementary concept word, protocol supplementary concept word, rare disease supplementary concept word, unique identifier, synonyms, population supplementary concept word, anatomy supplementary concept word] 119
87 ("Health-Related Quality of Life" or HRQL).mp. [mp=title, book title, abstract, original title, name of substance word, subject heading word, floating sub-heading word, keyword heading word, organism supplementary concept word, protocol supplementary concept word, rare disease supplementary concept word, unique identifier, synonyms, population supplementary concept word, anatomy supplementary concept word] 58107
88 ("global quality of life scale" or GQLS).mp. [mp=title, book title, abstract, original title, name of substance word, subject heading word, floating sub-heading word, keyword heading word, organism supplementary concept word, protocol supplementary concept word, rare disease supplementary concept word, unique identifier, synonyms, population supplementary concept word, anatomy supplementary concept word] 35
89 ("Nottingham health profile" or NHP).mp. [mp=title, book title, abstract, original title, name of substance word, subject heading word, floating sub-heading word, keyword heading word, organism supplementary concept word, protocol supplementary concept word, rare disease supplementary

concept word, unique identifier, synonyms, population supplementary concept word, anatomy supplementary concept word] 4306
90 ("12-Item Short Form Health Survey" or SF-12 or "8-Item Short Form Health Survey" or SF-8 or "36-Item Short Form Health Survey" or SF-36).mp. [mp=title, book title, abstract, original title, name of substance word, subject heading word, floating sub-heading word, keyword heading word, organism supplementary concept word, protocol supplementary concept word, rare disease supplementary concept word, unique identifier, synonyms, population supplementary concept word, anatomy supplementary concept word] 32332
91 ("Psychological General Well-Being Index" or PGWBI).mp. [mp=title, book title, abstract, original title, name of substance word, subject heading word, floating sub-heading word, keyword heading word, organism supplementary concept word, protocol supplementary concept word, rare disease supplementary concept word, unique identifier, synonyms, population supplementary concept word, anatomy supplementary concept word] 310
92 ("Assessment of Quality of Life" or AQoL).mp. [mp=title, book title, abstract, original title, name of substance word, subject heading word, floating sub-heading word, keyword heading word, organism supplementary concept word, protocol supplementary concept word, rare disease supplementary concept word, unique identifier, synonyms, population supplementary concept word, anatomy supplementary concept word] 2284
93 ("Control, Autonomy, Self-realization and Pleasure" or CASP).mp. [mp=title, book title, abstract, original title, name of substance word, subject heading word, floating sub-heading word, keyword heading word, organism supplementary concept word, protocol supplementary concept word, rare disease supplementary concept word, unique identifier, synonyms, population supplementary concept word, anatomy supplementary concept word] 2644
94 (EuroQol or EQ-5D).mp. [mp=title, book title, abstract, original title, name of substance word, subject heading word, floating sub-heading word, keyword heading word, organism supplementary concept word, protocol supplementary concept word, rare disease supplementary concept word, unique identifier, synonyms, population supplementary concept word, anatomy supplementary concept word] 15410
95 ("Visual analogue scale" or EQ-VAS).mp. [mp=title, book title, abstract, original title, name of substance word, subject heading word, floating sub-heading word, keyword heading word, organism supplementary concept word, protocol supplementary concept word, rare disease supplementary concept word, unique identifier, synonyms, population supplementary concept word, anatomy supplementary concept word] 31723
96 ("Patient-Reported Outcomes Measurement Information System" or PROMIS).mp. [mp=title, book title, abstract, original title, name of substance word, subject heading word, floating sub-heading word, keyword heading word, organism supplementary concept word, protocol supplementary concept word, rare disease supplementary concept word, unique identifier, synonyms, population supplementary concept word, anatomy supplementary concept word] 3925
97 "global health scale".mp. [mp=title, book title, abstract, original title, name of substance word, subject heading word, floating sub-heading word, keyword heading word, organism supplementary concept word, protocol supplementary concept word, rare disease supplementary concept word, unique identifier, synonyms, population supplementary concept word, anatomy supplementary concept word] 73
98 "EUROHIS-QOL 8-item index".mp. [mp=title, book title, abstract, original title, name of substance word, subject heading word, floating sub-heading word, keyword heading word, organism supplementary concept word, protocol supplementary concept word, rare disease supplementary concept word, unique

identifier, synonyms, population supplementary concept word, anatomy supplementary concept word] 18
99 ("Asthma Quality-of-Life Questionnaire" or AQLQ-M).mp. [mp=title, book title, abstract, original title, name of substance word, subject heading word, floating sub-heading word, keyword heading word, organism supplementary concept word, protocol supplementary concept word, rare disease supplementary concept word, unique identifier, synonyms, population supplementary concept word, anatomy supplementary concept word] 940
100 ("Caregiver Quality of Life Index-Cancer" or CQoLC).mp. [mp=title, book title, abstract, original title, name of substance word, subject heading word, floating sub-heading word, keyword heading word, organism supplementary concept word, protocol supplementary concept word, rare disease supplementary concept word, unique identifier, synonyms, population supplementary concept word, anatomy supplementary concept word] 78
101 ("Dermatology Life Quality Index" or DLQI).mp. [mp=title, book title, abstract, original title, name of substance word, subject heading word, floating sub-heading word, keyword heading word, organism supplementary concept word, protocol supplementary concept word, rare disease supplementary concept word, unique identifier, synonyms, population supplementary concept word, anatomy supplementary concept word] 3071
102 ("The European Organization for Research and Treatment of Cancer Quality of Life Questionnaire Core 30" or EORTC-QLQ-C30).mp. [mp=title, book title, abstract, original title, name of substance word, subject heading word, floating sub-heading word, keyword heading word, organism supplementary concept word, protocol supplementary concept word, rare disease supplementary concept word, unique identifier, synonyms, population supplementary concept word, anatomy supplementary concept word] 4035
103 ("Freiburg Quality of Life Assessment for Dermatitis" or FQLA-d).mp. [mp=title, book title, abstract, original title, name of substance word, subject heading word, floating sub-heading word, keyword heading word, organism supplementary concept word, protocol supplementary concept word, rare disease supplementary concept word, unique identifier, synonyms, population supplementary concept word, anatomy supplementary concept word] 0
104 ("Inflammatory Bowel Disease Questionnaire" or IBDQ).mp. [mp=title, book title, abstract, original title, name of substance word, subject heading word, floating sub-heading word, keyword heading word, organism supplementary concept word, protocol supplementary concept word, rare disease supplementary concept word, unique identifier, synonyms, population supplementary concept word, anatomy supplementary concept word] 698
105 ("Oral Health Impact Profile" or OHIP).mp. [mp=title, book title, abstract, original title, name of substance word, subject heading word, floating sub-heading word, keyword heading word, organism supplementary concept word, protocol supplementary concept word, rare disease supplementary concept word, unique identifier, synonyms, population supplementary concept word, anatomy supplementary concept word] 2482
106 ("Oral Impacts on Daily Performance" or OIDP).mp. [mp=title, book title, abstract, original title, name of substance word, subject heading word, floating sub-heading word, keyword heading word, organism supplementary concept word, protocol supplementary concept word, rare disease supplementary concept word, unique identifier, synonyms, population supplementary concept word, anatomy supplementary concept word] 344
107 ReTransQol.mp. [mp=title, book title, abstract, original title, name of substance word, subject heading word, floating sub-heading word, keyword heading word, organism supplementary concept word, protocol supplementary concept word, rare disease supplementary concept word, unique identifier, synonyms, population supplementary concept word, anatomy supplementary concept word] 5

108 ("Short Inflammatory Bowel Disease Questionnaire" or SIBDQ).mp. [mp=title, book title, abstract, original title, name of substance word, subject heading word, floating sub-heading word, keyword heading word, organism supplementary concept word, protocol supplementary concept word, rare disease supplementary concept word, unique identifier, synonyms, population supplementary concept word, anatomy supplementary concept word] 174
109 "Vision Functioning and Quality of Life".mp. [mp=title, book title, abstract, original title, name of substance word, subject heading word, floating sub-heading word, keyword heading word, organism supplementary concept word, protocol supplementary concept word, rare disease supplementary concept word, unique identifier, synonyms, population supplementary concept word, anatomy supplementary concept word] 1
110 ("sickness impact profile" or SIP).mp. [mp=title, book title, abstract, original title, name of substance word, subject heading word, floating sub-heading word, keyword heading word, organism supplementary concept word, protocol supplementary concept word, rare disease supplementary concept word, unique identifier, synonyms, population supplementary concept word, anatomy supplementary concept word] 11146
111 ("quality of well-being" or QWB).mp. [mp=title, book title, abstract, original title, name of substance word, subject heading word, floating sub-heading word, keyword heading word, organism supplementary concept word, protocol supplementary concept word, rare disease supplementary concept word, unique identifier, synonyms, population supplementary concept word, anatomy supplementary concept word] 518
112 ("McMaster Health Index Questionnaire" or MHIQ).mp. [mp=title, book title, abstract, original title, name of substance word, subject heading word, floating sub-heading word, keyword heading word, organism supplementary concept word, protocol supplementary concept word, rare disease supplementary concept word, unique identifier, synonyms, population supplementary concept word, anatomy supplementary concept word] 17
113 ("General Health Rating Index" or GHRI).mp. [mp=title, book title, abstract, original title, name of substance word, subject heading word, floating sub-heading word, keyword heading word, organism supplementary concept word, protocol supplementary concept word, rare disease supplementary concept word, unique identifier, synonyms, population supplementary concept word, anatomy supplementary concept word] 25
114 ("Spiritual Well- being scale" or Facit-Sp).mp. [mp=title, book title, abstract, original title, name of substance word, subject heading word, floating sub-heading word, keyword heading word, organism supplementary concept word, protocol supplementary concept word, rare disease supplementary concept word, unique identifier, synonyms, population supplementary concept word, anatomy supplementary concept word] 484
115 "Thriving of Older People Assessment Scale".mp. [mp=title, book title, abstract, original title, name of substance word, subject heading word, floating sub-heading word, keyword heading word, organism supplementary concept word, protocol supplementary concept word, rare disease supplementary concept word, unique identifier, synonyms, population supplementary concept word, anatomy supplementary concept word] 4
116 ("Spiritual Distress Assessment Tool" or SDAT).mp. [mp=title, book title, abstract, original title, name of substance word, subject heading word, floating sub-heading word, keyword heading word, organism supplementary concept word, protocol supplementary concept word, rare disease supplementary concept word, unique identifier, synonyms, population supplementary concept word, anatomy supplementary concept word] 660
117 ("Positive Valuation of Life Scale" or "Positive VOL").mp. [mp=title, book title, abstract, original title, name of substance word, subject heading word, floating sub-heading word, keyword heading word,

organism supplementary concept word, protocol supplementary concept word, rare disease supplementary concept word, unique identifier, synonyms, population supplementary concept word, anatomy supplementary concept word] 4

118 ("Philadelphia Geriatric Center Morale Scale" or PGCMS).mp. [mp=title, book title, abstract, original title, name of substance word, subject heading word, floating sub-heading word, keyword heading word, organism supplementary concept word, protocol supplementary concept word, rare disease supplementary concept word, unique identifier, synonyms, population supplementary concept word, anatomy supplementary concept word] 116

119 ("Herth Hope Index" or HHI).mp. [mp=title, book title, abstract, original title, name of substance word, subject heading word, floating sub-heading word, keyword heading word, organism supplementary concept word, protocol supplementary concept word, rare disease supplementary concept word, unique identifier, synonyms, population supplementary concept word, anatomy supplementary concept word] 519

120 ("Ideas about Long-Standing Health Problems" or ILSHP).mp. [mp=title, book title, abstract, original title, name of substance word, subject heading word, floating sub-heading word, keyword heading word, organism supplementary concept word, protocol supplementary concept word, rare disease supplementary concept word, unique identifier, synonyms, population supplementary concept word, anatomy supplementary concept word] 1

121 "Flourishing Scale".mp. [mp=title, book title, abstract, original title, name of substance word, subject heading word, floating sub-heading word, keyword heading word, organism supplementary concept word, protocol supplementary concept word, rare disease supplementary concept word, unique identifier, synonyms, population supplementary concept word, anatomy supplementary concept word]0

122 ("Nursing Home Adjustment Scale" or NHAS).mp. [mp=title, book title, abstract, original title, name of substance word, subject heading word, floating sub-heading word, keyword heading word, organism supplementary concept word, protocol supplementary concept word, rare disease supplementary concept word, unique identifier, synonyms, population supplementary concept word, anatomy supplementary concept word] 196

123 "PERMA Profiler".mp. [mp=title, book title, abstract, original title, name of substance word, subject heading word, floating sub-heading word, keyword heading word, organism supplementary concept word, protocol supplementary concept word, rare disease supplementary concept word, unique identifier, synonyms, population supplementary concept word, anatomy supplementary concept word] 29

124 ("International Wellbeing Index" or "Personal Wellbeing Index").mp. [mp=title, book title, abstract, original title, name of substance word, subject heading word, floating sub-heading word, keyword heading word, organism supplementary concept word, protocol supplementary concept word, rare disease supplementary concept word, unique identifier, synonyms, population supplementary concept word, anatomy supplementary concept word] 117

125 ("ICEpop CAPability measure for older people" or ICECAP-O).mp. [mp=title, book title, abstract, original title, name of substance word, subject heading word, floating sub-heading word, keyword heading word, organism supplementary concept word, protocol supplementary concept word, rare disease supplementary concept word, unique identifier, synonyms, population supplementary concept word, anatomy supplementary concept word] 84

126 ("Quality of Life questionnaire for Dementia" or QOL-D).mp. [mp=title, book title, abstract, original title, name of substance word, subject heading word, floating sub-heading word, keyword heading word, organism supplementary concept word, protocol supplementary concept word, rare disease supplementary concept word, unique identifier, synonyms, population supplementary concept word, anatomy supplementary concept word] 33

127 QUALIDEM.mp. [mp=title, book title, abstract, original title, name of substance word, subject heading word, floating sub-heading word, keyword heading word, organism supplementary concept word, protocol supplementary concept word, rare disease supplementary concept word, unique identifier, synonyms, population supplementary concept word, anatomy supplementary concept word] 75

128 ("Quality of life-Alzehimer´s Disease scale" or QOL-AD).mp. [mp=title, book title, abstract, original title, name of substance word, subject heading word, floating sub-heading word, keyword heading word, organism supplementary concept word, protocol supplementary concept word, rare disease supplementary concept word, unique identifier, synonyms, population supplementary concept word, anatomy supplementary concept word] 259

129 ("Dementia Quality of Life" or DQOL).mp. [mp=title, book title, abstract, original title, name of substance word, subject heading word, floating sub-heading word, keyword heading word, organism supplementary concept word, protocol supplementary concept word, rare disease supplementary concept word, unique identifier, synonyms, population supplementary concept word, anatomy supplementary concept word] 238

130 EQVILPI.mp. [mp=title, book title, abstract, original title, name of substance word, subject heading word, floating sub-heading word, keyword heading word, organism supplementary concept word, protocol supplementary concept word, rare disease supplementary concept word, unique identifier, synonyms, population supplementary concept word, anatomy supplementary concept word] 0

131 ("Minnesota Living with Heart Failure Questionnaire" or MLHFQ).mp. [mp=title, book title, abstract, original title, name of substance word, subject heading word, floating sub-heading word, keyword heading word, organism supplementary concept word, protocol supplementary concept word, rare disease supplementary concept word, unique identifier, synonyms, population supplementary concept word, anatomy supplementary concept word] 865

132 82 or 83 or 84 or 85 or 86 or 87 or 88 or 89 or 90 or 91 or 92 or 93 or 94 or 95 or 96 or 97 or 98 or 99 or 100 or 101 or 102 or 103 or 104 or 105 or 106 or 107 or 108 or 109 or 110 or 111 or 112 or 113 or 114 or 115 or 116 or 117 or 118 or 119 or 120 or 121 or 122 or 123 or 124 or 125 or 126 or 127 or 128 or 129 or 130 or 131 496321

133 81 and 132 309160

134 (bangladesh* or bengal* or bangal* or bhutan* or india* or indonesia* or korea* or maldives* or maldivian* or indian ocean or myanmar* or burma* or burmese or nepal* or sri lanka* or ceylon* or thai* or timor leste* or east timor* or timorese* or southeastern asia* or south eastern asia* or southeast asia* or south east asia* or developing countr* or developing nation* or developing population* or developing world or less developed countr* or less developed nation* or less developed world or lesser developed countr* or lesser developed nation* or lesser developed world or under developed countr* or under developed nation* or under developed world or underdeveloped countr* or underdeveloped nation* or underdeveloped world or middle income countr* or middle income nation* or middle income population* or low income countr* or low income nation* or low income population* or lower income countr* or lower income nation* or lower income population* or underserved countr* or underserved nation* or underserved population* or under served population* or under served nation* or under served population* or deprived countr* or deprived population* or high burden countr* or high burden nation* or countdown countr* or countdown nation* or poor countr* or poor nation* or poor population* or poor world or poorer countr* or poorer nation* or poorer population* or poorer world or developing econom* or less developed econom* or underdeveloped econom* or under developed econom* or middle income econom* or low income econom* or lower income econom* or low gdp or low gnp or low gross domestic or low gross national or lower gdp or lower gnp or lower gross domestic or lower gross national or lmic or lmics or third world or lami countr* or transitional countr* or

emerging econom* or emerging nation*).mp. [mp=title, book title, abstract, original title, name of substance word, subject heading word, floating sub-heading word, keyword heading word, organism supplementary concept word, protocol supplementary concept word, rare disease supplementary concept word, unique identifier, synonyms, population supplementary concept word, anatomy supplementary concept word] 671917

135 133 and 134 9275

136 (bangladesh* or bengal* or bangal* or bhutan* or india* or indonesia* or korea* or maldives* or maldivian* or indian ocean or myanmar* or burma* or burmese or nepal* or sri lanka* or ceylon* or thai* or timor leste* or east timor* or timorese* or southeastern asia* or south eastern asia* or southeast asia* or south east asia*).mp. [mp=title, book title, abstract, original title, name of substance word, subject heading word, floating sub-heading word, keyword heading word, organism supplementary concept word, protocol supplementary concept word, rare disease supplementary concept word, unique identifier, synonyms, population supplementary concept word, anatomy supplementary concept word] 508419

137 133 and 136 6952

138 limit 137 to dt=20230501-20240830 [May 1st, 2023 to August 30th, 2024]1184

References

1. Alfian SD*, et al.* Emotional distress is associated with lower health-related quality of life among patients with diabetes using antihypertensive and/or Antihyperlipidemic medications: A multicenter study in Indonesia. *Therapeutics and clinical risk management*, 1333-1342 (2021).
2. Banerjee S, Mukherjee A, Bhattacharyya B, Mohanakumar KP, Biswas A. Quality of life and concerns of Parkinson's disease patients and their caregivers during COVID-19 pandemic: an Indian study. *Annals of Indian Academy of Neurology* **25**, 676-682 (2022).
3. Barne S, Haral EM. Cardiopulmonary Functions and Quality of Life in Patients with Pulmonary Tuberculosis: A Cross-sectional Study from Pune, Maharashtra, India. *Journal of Clinical & Diagnostic Research* **18**, (2024).
4. Basu G, Nandi D, Biswas S, Roy SK. Quality of life and depression among diabetic patients attending the lifestyle clinic of a teaching hospital, West Bengal. *Journal of Family Medicine and Primary Care* **10**, 321-326 (2021).
5. Dejvorakul S, Kumar R, Srirojanakul S, Panupichit N, Somrongthong R. Factors predicted with quality of life among hemodialysis patients in private hospital of Thailand. *Hospital Practice* **47**, 254-258 (2019).
6. Fadhil A*, et al.* Effect of agomelatine and sertraline on patients with major depressive disorders and chronic kidney disease: a randomized controlled trial. *Journal of Southeast Asian Medical Research* **6**, e0127-e0127 (2022).
7. Gupta A*, et al.* Association between Vision-related Quality of Life and Mental Health Status among Glaucoma Patients in a Tertiary Care Hospital, Uttar Pradesh, India: A Cross-sectional Study. *Journal of Clinical & Diagnostic Research* **18**, (2024).
8. Hanspal I, Fathima FN, Kedlaya PG. Social impact of end-stage renal disease requiring hemodialysis among patients with type-2 diabetes and their caregivers in Bengaluru, Karnataka. *Indian Journal of Community Medicine* **46**, 626-630 (2021).
9. Hussain S, Habib A, Najmi AK. Anemia prevalence and its impact on health-related quality of life in Indian diabetic kidney disease patients: evidence from a cross-sectional study. *Journal of Evidence-Based Medicine* **12**, 243-252 (2019).
10. Koesoemadinata R*, et al.* Educational counselling of patients with combined TB and diabetes mellitus: a randomised trial. *Public health action* **11**, 202-208 (2021).
11. Komariah A, Rochmawati E. The Effect of Listening to the Holy Qur’an and a Back Massage on Fatigue and Quality of Life for Participants Undergoing Hemodialysis: A Quasi-Experimental Study. *Journal of religion and health* **62**, 4334-4346 (2023).
12. Kumar SB, Karthikeyan B, Nair SV, Ramasamy A, Khan S, Periasamy S. A study of factors affecting Dialysis recovery time in Haemodialysis patients in India. *Indian journal of nephrology* **31**, 460-466 (2021).
13. Kunwar D, Kunwar R, Shrestha B, Amatya R, Risal A. Depression and quality of life among the chronic kidney disease patients. (2020).
14. Kuptniratsaikul V, Kovindha A, Suethanapornkul S, Manimmanakorn N, Archongka Y. Complications during the rehabilitation period in Thai patients with stroke: a multicenter prospective study. *American journal of physical medicine & rehabilitation* **88**, 92-99 (2009).
15. Manimmanakorn NM, Vichiansiri R, Nuntharuksa C, Permsirivanich W, Vilai Kuptniratsaikul M. Quality of life after stroke rehabilitation among urban vs. rural patients in Thailand. *J Med Assoc Thai* **9**, 394-399 (2008).

16. Mishra R*, et al.* Impact of metformin therapy on health-related quality of life outcomes in tuberculosis patients with diabetes mellitus in India: A prospective study. *International Journal of Clinical Practice* **75**, e13864 (2021).
17. Modi GK*, et al.* Nonmedical factors and health-related quality of life in CKD in India. *Clinical Journal of the American Society of Nephrology* **15**, 191-199 (2020).
18. Murali R, Sathyanarayana D, Muthusethupathy M. Assessment of quality of life in chronic kidney disease patients using the kidney disease quality of life-short formtm questionnaire in indian population: A community based study. *Asian Journal of Pharmaceutical and Clinical Research* **8**, 271-274 (2015).
19. Patel B, Oza B, Patel K, Malhotra S, Patel V. Health related quality of life in type-2 diabetic patients in Western India using World Health Organization Quality of Life–BREF and appraisal of diabetes scale. *International journal of diabetes in developing countries* **34**, 100-107 (2014).
20. Prakash B, Krishnaveni YS, Narayanamurthy MR. Mental Health Status among Patients with Breast Cancer Attending a Tertiary Care Cancer Hospital in Mysuru, Karnataka, India. *Indian Journal of Public Health Research & Development* **10**, (2019).
21. Prasad M, Kumar VS. Assesment of socio - economic factors on medication adherance and quality of life in patients with hypertension and type 2 diabetes. *NeuroQuantology* **20**, 2671-2682 (2022).
22. Rahman R, Mariam L, Su R, Malhotra C, Ozdemir S. Quality of life and its predictors among patients with metastatic cancer in Bangladesh: the APPROACH survey. *BMC Palliative Care* **23**, 2 (2024).
23. Ranabhat K, Khanal P, Mishra SR, Khanal A, Tripathi S, Sigdel MR. Health related quality of life among haemodialysis and kidney transplant recipients from Nepal: a cross sectional study using WHOQOL-BREF. *BMC nephrology* **21**, 1-8 (2020).
24. Saisunantararom W, Cheawchanwattana A, Kanjanabuch T, Buranapatana M, Chanthapasa K. Associations among spirituality, health-related quality of life, and depression in pre-dialysis chronic kidney disease patients: An exploratory analysis in thai buddhist patients. *Religions* **6**, 1249-1262 (2015).
25. Singh V, Kumari G, Chhajer B, Vijayasimha M. Efficacy of Enhanced External Counter Pulsation on Clinical Parameters and Health-related Quality of Life in Coronary Heart Disease patients with Diabetes Mellitus. *Biomedical and Pharmacology Journal* **12**, 1937-1949 (2019).
26. Singh G, Mahajan N, Abrol S, Raina A. Anxiety and depression are common in rheumatoid arthritis and correlate with poor quality of life in Indian patients. *Reumatologia/Rheumatology* **59**, 386-393 (2021).
27. Sridhar T*, et al.* A study evaluating the aspects of stroke-specific quality of life and severity in hypertensive stroke patients. *Asian Journal of Pharmaceutical Research and Health Care* **16**, 216-224 (2024).
28. Sudarisan SSP, Abraham B, George C. Prevalence, correlates of depression, and its impact on quality of life of cancer patients attending a palliative care setting in South India. *Psycho-oncology* **28**, 1308-1313 (2019).
29. Sukcharoen N, Hounnaklang N, Tantirattanakulchai P, Win N. Hierarchical Regression of Diabetes Self-Management and Health Related Quality of Life among Older Adults Patients with Type 2 Diabetes Mellitus. *International Journal of Nursing Education* **16**, (2024).
30. Thanakiatpinyo T*, et al.* The efficacy of traditional Thai massage in decreasing spasticity in elderly stroke patients. *Clinical interventions in aging*, 1311-1319 (2014).
31. Thancharoen O, Waleekhachonloet O, Limwattananon C, Anutrakulchai S. Cognitive impairment, quality of life and healthcare utilization in patients with chronic kidney disease stages 3 to 5. *Nephrology* **25**, 625-633 (2020).

32. Tungsirikoon N, Howteerakul N, Suwannapong N, Rawdaree P. An Audit of Diabetes-Dependent Quality of Life and Glycemic Control Among Type 2 Diabetes Patients in a Tertiary Hospital in Bangkok: A Hospital-based Cross-sectional Study. *Journal of Health Research* **37**, 10 (2023).

33. Yapa HE, Purtell L, Chambers S, Bonner A. Factors influencing health-related quality of life in people with chronic kidney disease: A structural equation modelling approach. *Journal of Clinical Nursing* **32**, 3445-3455 (2023).

34. Gautam P, Dahal P, Paul D, Alam K. Health-Related Knowledge Attitude Practice and Quality of Life Among Diabetic Hypertensive Patients in Eastern Nepal. *Pharmacophore* **14**, 11-18 (2023).
